# 

**Published:** 1846-01

**Authors:** 


### THE MEDICO-CHIRURGICAL REVIEW, AND JOURNAL OF PRACTICAL MEDICINE.

v. [44]

NEW SERIES.

VOL. III.

Nothing to index

OCTOBER 1, 1845, TO MARCH 31, 1846.

Surgeon General's Office LIBRARY 57484

LONDON:

SAMUEL HIGHLEY, 32, FLEET STREET,

1846.

wl
ME 75T

PRINTED BY F. HAYDEN,

Little College Street, Westminster.

### INDEX.

A.

Abdomen, diagnosis of inflammation of 51
Abdomen, case of tumour of........ 325
Abortion from rheumatism of uterus 346
Abscess, hepatic.................. 66
Abscess, hydatid.................. 327
Abscess, mammary .............. 223
Abscess, metastatic ............. 240
Abscess, opening of .............. 222
Abscess of pharynx .............. 225
Abscess, psoas (position in)........ 75
Abscess, urinary.................. 119
Acne treated by caustics .......... 542
Aconite, Fleming on .............. 248
Aconite, physiological action of .... 249
Aconite, poisoning by ............ 256
Aconite, therapeutical action........ 255
Aconite, mode of administering .... 259
Adventitious productions .......... 477
Air-passages, Kermes in diseases of.. 543
Albuminuria, fatality of........ .... 129
Albuminuria, treatment of ........ 162
Albuminuria, Lebert on............. 239
Albuminuria, a cause of pericarditis.. 448
Algæ, British, Hassal on .......... 217
Algeria, disease of liver in.......... 63
Algeria, phthisis in ............ 67, 139
Alligators, Dr. Johnson on ........ 10
Amputation of hip-joint .......... 277
Anatomy, comparative, Cuvier's .... 285
Anatomy, comparative, Wagner's.... 401
Aneurism, brachial .............. 358
Aneurism, popliteal, compression in 196
Aneurism without symptoms ...... 452
Aneurism of bones....... ....... 559
Ani, fistula ...................... 228
Animal heat, Rogers on .......... 427
Animal heat, Prout on ............ 535
Animalcules, Klencke on .......... 261
Antidote, charcoal as an .......... 332
Anus, tissue of the................ 228
Aphthæ in animals................ 461
Apoplexy, meningeal.............. 198
Apoplexy, bleeding in ........... 53
Apoplexy, nervous................ 189
Apothecaries' Society, Address of.... 282
Arachnoid, extravasation of blood into 198
Arnott's therapeutical enquiry ...... 540
Arteries, wounds of .............. 226
Arteries, pulmonary, obstruction of.. 437
Artery, temporal, compression of.... 225
Assimilation, Prout on .......... 535
Asthma, Favell on the grinders'...... 533
Auscultation, Hughes on .......... 279

B.

Ballard, materia medica .. ........ 163
Baly on prisons ................... 208
Barker on aneurism ............. 452
Barlow on dropsies................ 126
Beck on nerves of uterus .......... 526
Bell on the microscope . .......... 321
Bennett, Dr. J. H. on diseases of the uterus .............................. 337
Bennett, Dr. J. R. on furuncular eruption ......... ................. 334
Bile, vitiation of.................. 26
Berard on hæmatocele ............ 561
Birds, vocal organs of ............ 405
Birds, tegumentary muscles of ...... 407
Bishop on stammering ............ 322
Black-Hole, the .................. 12
Bladder, inversion of the .......... 534
Bladder, wound of the ............ 359
Blake on formation of teeth ........ 300
Bland on chlorosis.. ............. 557
Blood, analysis of the.............. 152
Blood, organization of extravasated.. 201
Blood, condition of, in inflammation 236
Blood, hydatids in the ............ 268
Blood, formation of red particles of.. 428
Bone, mechanical structure of ...... 429
Bones, pulsating tumours of........ 433
Bones, cancer of.................. 492
Bones, aneurisms of .......... 433, 559
Bowels, cases of obstruction of the 205, 329
Brain, bleeding in disease of ........ 53
Brain, hydatids of the ............ 264
Brain, Todd on anatomy of the...... 285
Brain, temperature in disease of .... 417
Brain, diseases of, in animals ...... 474
Brain, issues in disease of ........ 187
Bread, coarse, advantages of........ 382
Breast, abscess of ................ 223
Bright's disease, see albuminaria ....
Bronchitis, Kermes in ............ 543
Bronchitis, temperature in ........ 418
Brown on scarlatina .............. 540
Bubo, Colles on................... 232

C.

Cachexia in animals ... 471
Cæsarean operation, cases of ... 356
Calomel, employment of, in India .. 369
Cancer of the stomach ... 181
Cancer, Lebert on structure of ... 244
Cancer of the lip ... 245
Cancer, Walshe on ... 475
Cancer, nosological position of ... 476
Cancer, chemistry of ... 483
Cancer, proximate cause of ... 483
Cancer, origin in the blood ... 484
Cancer, vascular supply to ... 486
Cancer, process of decay ... 487
Cancer, dissemination of ... 488
Cancer, of the various organs ... 491
Cancer, symptoms of ... 492
Cancer, terminations ... 493
Cancer, frequency and duration ... 494
Cancer, mortality and etiology ... 497
Cancer, diagnosis ... 496
Cancer, constitutional nature of ... 500
Cancer, treatment of ... 501
Cancer, compression of ... 504
Cancer, question of operating in ... 506
Capillaries, condition in inflammation 235
Cataplasms, cold ... 558
Catarrh in animals ... 471
Catarrh, gouty ... 87
Catteloup on hepatitis ... 65
Cement of the teeth, Owen on the .. 296
Cement, mode of formation ... 299
Cephalhæmatoma, West on ... 438
Chancre, treatment of ... 457
Charcoal, animal, as an antidote ... 332
Chelius on hernia ... 508
Chemistry, Gregory's outlines ... 286
Children, the crowing disease of ... 55
Children, defective power of generating heat ... 133, 410
Children, normal temperature ... 409
Children, temperature in disease, 411, 424
Children, œdema of new-born ... 422
Children, Trousseau on the eruptions of ... 549
China, diseases of ... 15
Chlorosis of adults ... 557
Cholera, Copland on ... 146
Cholera, contagion of the ... 148, 365
Cholera, insect origin of ... 261
Cholera, treatment of ... 363
Cholera, turpentine in ... 421
Chorea, treatment of ... 129
Chorea, temperature in ... 420
Clark's case of urethro-plastie ... 440
Climate, Johnson on ... 22
Climate, Levy on ... 137
Club-foot, varieties of ... 390
Colchicum, action of ... 93
Cold, resistance of, according to temperament ... 133
Cold, non-resistance of children to 133, 410
Coles on spinal affections ... 71
Colles' lectures on surgery ... 221
Colloid growths, Walshe on ... 481
Colombat on diseases of women ... 337
Colon, obstruction of the ... 205
Colostrum of the cow ... 204
Concussion, Colles on ... 224
Confervæ, uses of ... 219
Constipation, Schoenlein on ... 181
Cooper on fracture of cervix femoris 123
Copland's Dictionary ... 143
Country, signs of a unhealthy ... 19
Cox on amputation at the hip-joint.. 277
Cranium, ossification of the ... 430
Cranium, fracture of basis of ... 547
Crosse on inversion of the bladder .. 534
Croton oil, employment of, in India.. 364
Croup, turpentine in ... 418
Curling on hypertrophy of the fingers 436
Cuvier's comparative anatomy ... 285
Cystoid tumours distinguished from cancer ... 496

D.

Davy on collostrum of cow ... 204
Dubois on pregnancy ... 562
Deafness, kermes in ... 543
Deformities, Tamplin on ... 388
Delirium tremens, opium in ... 374
Dendy on abdominal tumour ... 325
Dendy on ulcer of stomach ... 331
Dentine, Owen on the ... 292
Dentine, vascular and unvascular ... 295
Dentine, mode of formation of ... 297
Dentition in animals ... 461
Diabetes, treatment of ... 163, 183
Diabetes in animals ... 469
Diet, vegetable, advantages of ... 379
Diet, vegetable, in disease ... 384
Digestion, organs of, temperature in diseases of ... 415
Digestion, organs of, diseases of, in animals ... 462
Disease, insect, origin of ... 261
Diseases of animals ... 461
Dropsy, from obstruction of heart or lungs ... 126
Dropsy, temperature in ... 415
Dropsy, ovarian, cure of ... 531
Duodenum, malformation ... 455
Dysentery from hepatitis ... 65
Dysentery in India ... 369
Dysentery, temperature in ... 416
Dyspepsia, vegetable diet in ... 384

E.

Ear, discharge from, in fracture of the cranium ... 547
Echinococcus hominis ... 194
Elbow, excision of the ... 545
Enamel of the teeth, formation of ... 299
Encephaloid, Walshe on ... 478
Enchondroma, Walshe on ... 498
Enteritis, treatment of ... 49

Enteritis, temperature in .......... 415
Enteritis in animals .............. 463
Entozoa, Klencke on.......... 261, 272
Epilepsy, quinine in .............. 544
Erectile tumour distinguished from cancer.......................... 498
Eruptions, syphilitic.............. 232
Eruptions of children.............. 549
Erysipelas, cold affusion in ........ 185
Erysipelas, Colles on .............. 222
Erysipelas of the scalp ............ 224
Exanthemata in animals............ 466
Exanthemata, influence of menses on 186
Eye injured by artificial light ...... 140
Eyelids, tumors of the ............ 224

F.

Face, black secretion of the ........ 453
Face, eruptions of, in children...... 549
Farcy, Heusinger on .............. 472
Favell on grinder's asthma ........ 533
Feet, deformities of the............ 390
Females, Colombat on Diseases of .. 340
Femur, Cooper on fracture of the cervix .......................... 123
Femur, Fergusson on excision of the head of ...................... 450
Fergusson on cleft palate .......... 431
Fergusson on excision of head of the femur ...................... 450
Fever, venereal hectic ............ 230
Fever, puerperal, treatment of...... 355
Fever, Indian remittent............ 366
Fever, Indian intermittent ........ 369
Fever, temperature in intermittent .. 411
Fever, temperature in typhoid...... 412
Fibrous growths distinguished from cancer .......................... 496
Fingers, hypertrophy of............ 436
Fishes, sympathetic system of...... 404
Fishes, swimming bladder of ...... 407
Fishes, Owen on the teeth of ...... 304
Fistula in ano.... ................ 228
Fistula, urinary.................. 360
Fleming on aconite .............. 249
Fæces, accumulation of............ 181
Fœtation, extra-uterine............ 121
Food, vegetable, advantages of...... 379
Food, animal, injuriousness of...... 383
Forceps, the, in tedious labour...... 524
France, med. reform in ............ 283
Fruits and farinacea, Smith on...... 378
Fumigations, mercurial.......... 455
Furuncular eruptions ............ 334

G.

Galvanism in lumbago, &c. ........ 553
Gangrene, dry, in India .......... 376
Gangrene, temperature in ........ 421
Garrod's Materia Medica .......... 163
Garrod on charcoal as an antidote .. 332
Glanders, Heusinger on .......... 472
Gleet, stricture in ................ 117
Gonorrhœa, Colles on ............ 229
Gout, Robertson on .............. 78
Gout, constitutional nature of. .... 79
Gout, connexion with plethora ..... 81
Gout, predisposing causes of ...... 82
Gout, exciting causes of ........... 84
Gout, proximate cause of.......... 86
Gout, distinguished from rheumatism, 90
Gout, treatment ................ 92
Gregory's Outlines of Chemistry.... 286
Gregory's translation on magnetism, 536
Griffin's medical problems ......... 49

H.

Hæmatocele, spontaneous.......... 561
Hæmoptysis, Griffin on .......... 59
Hæmorrhoids in India ............ 375
Haspel on hyperæmia of the liver ... 163
Hassal on British algæ ............ 217
Heart, aconite in diseases of the .... 257
Heart, sounds of the.............. 281
Heart, malformations of the........ 403
Heart, temperature in diseases of the, 415
Henderson on homœopathy ........ 538
Hepatitis in Algeria .............. 65
Hernia, Belladonna in strangulated.. 357
Hernia in animals ................ 464
Hernia, Teale and South on ....... 508
Hernia, operation for, without opening the sac ...................... 508
Hernia, management of omentum in, 511
Hernia distinguished from hydrocele, 514
Hernia, operation on irreducible .... 329
Hernia in females ................ 515
Herpes treated by caustics ........ 542
Heusinger on comparative pathology, 459
Hewett on extravasation into the arachnoid ...................... 198
Hip-joint, amputation at the ...... 277
Hip-joint, contraction of the....... 398
Hip-joint, excision of the.......... 450
Homœopathy, Henderson and Sampson on.......................... 538
Hospitals, construction of ventilation of ........................... 140
Hughes on auscultation............ 279
Hunter, John, on formation of the teeth ........................ 300
Hydarthrosis, treatment of ........ 554
Hydatids, Klencke on ............ 264
Hydatids of various organs ........ 265
Hydatids, modes of entering the frame 269
Hydatids, Thompson on .......... 327
Hydrocele distinguished from hernia, 514
Hydrocele, iodine injections in ..... 554
Hydrocephalus, intermittents in .... 187
Hygiene, Levy on ................ 130

I.

Ileus, inflammatory.............. 180
India, preservation of health in .... 18
India, Macgregor on diseases of .... 363
Inflammation, intimate changes in .. 235

Insanity, pathology of ........... 441
Insanity, narcotics in ............. 287
Intestinal canal, obstruction of, 205, 329
Intestinal canal, diseases of, in animals 464
Intestinal concretions in animals. .. 462
Intestines, small structure and perforation of ...................... 331
Iodine, injections of ............. 554
Iron in menorrhagia ............. 544
Issues of the scalp ................ 187
Itch as a cause of disease .......... 189
Itch in animals .................. 466

J.

Jaundice, coma in ................ 54
Jaundice, slow pulse in ............ 179
Jaundice, treatment of ........ 179, 373
Jaw, contraction of the............ 400
Jejunum, strangulation of the ...... 331
Johnson, Dr. James, biography of... 1
Johnson on Tropical Climates ...... 22
Johnson's Essay on Indigestion ..... 40
Johnson's Change of Air .......... 41
Joints, excision of .............450, 545
Joints, treatment of dropsy of the .. 554

K.

Kermes mineral, Herpin on ........ 543
Kidney, obliteration of the ........ 128
Kidney, scrofula of the ............ 183
Kidney, condition of, in Bright's disease ......................... 239
Kidney, supposed tumour of ....... 325
Klencke on entozoary animalcules .. 261
Knee, contraction of.......... 396, 398
Knee, scrofulous disease of ........ 396
Knock-knee, Tamplin on .......... 393

L.

Labour, with rigid os uteri ........ 120
Labour, influence of rheumatism on.. 347
Labour, abrasion of cervix in ...... 350
Labour, process of natural ........ 521
Labour, management of tedious ..... 523
Labour, effects of despondency on .. 523
Labour, laborious ................ 524
Lallemand on stricture ............ 97
Laryngismus stridulus ............ 55
Laryngitis, Kermes in ............ 543
Laryngotomy, case of ............ 324
Larynx of birds .................. 405
Laugier on fracture of basis ........ 547
Lebert's Pathological Physiology .... 235
Legs, deformities of the............ 395
Lepra, treatment of .............. 129
Leroy on stricture ................ 97
Leucorrhœa from ulceration........ 349
Lever on rigid os uteri ............ 120
Levy on hygiene ................. 130
Lip, Lebert on cancer of the ....... 245
Lithic acid diathesis .............. 160
Lithotomy, new operation ......... 361
Liver, Johnson on diseases of ...... 24
Liver diseases in Algeria .......... 63
Liver, abscess of ................. 6[illegible]
Liver, fatty degeneration of........ 6[illegible]
Liver, hydatids of the ............ 266
Liver diseases in India ............ 37[illegible]
Liver, diseases of, in animals ...... 464
Longevity, favoured by vegetable food 386
Lumbago, galvanism in............ 553
Lungs, hydatids of ............... 267
Lupus, Stedman on .............. 336

M.

Macgregor on diseases of India...... 363
Madeira, Dr. Johnson on .......... 7
Magnetism, Reichenbach on........ 536
Malaria, nature and effects of ...... 134
Malgaigne on diagnosis of hernia.... 516
Measles, temperature in .......... 415
Medical reform .................. 282
Medical Society of London, Transactions of ......................... 321
Medicines, modus operandi of.. 165, 190
Medico-Chirurgical Review, history of ......................... 34
Medico-Chirurgical Transactions, 191, 431
Meigs on diseases of women........ 357
Melanosis distinguished from cancer, 499
Meningitis, temperature in ........ 416
Menorrhagia, iron in.............. 544
Menstruation in Venezuela ........ 338
Mercury, mode of administering .... 231
Mercury, fumigation of ........... 455
Mercury in vapour baths........... 455
Microscope, Bell on advantages of .. 321
Millbank Penitentiary, mortality at.. 208
Mountains, physiological effects of ascending ..................... 134
Mucus, characters of.............. 238
Murphy on parturition............ 516
Muscle, intimate structure of....... 430

N.

Narcotics, operation of ............ 167
Negro, structure of skin of ......... 429
Nelaton on aneurism of bones ...... 559
Nephritis, albuminous ........ 239, 240
Neuralgia, gouty ................ 88
Neuralgia, aconite in.............. 256
Nerves, the fifth pair of, affection of.. 438
Nervous system, diseases of, intermittence of ...................... 187
Nervous system, diseases of, temperature in ....................... 416
Numerical method, the............ 62
Nutrition by non-azotized principles, 381

O.

Odontography, Owen's ........... 289
Œdema of new-born children ...... 422
Oldham on extra-uterine fœtation .. 121
Omentum, management of, in hernia 511
Ophthalmia, puerperal ........... 437
Opium, therapeutical effects of ..... 57
Opium in delirium tremens ........ 374
Orbit, gun-shot wound of.......... 359

[illegible]riental-voyager, Dr. Johnson's .... 5
[illegible]ris cancrum, temperature in ...... 421
[illegible]variotomy, Southam's case of ..... 531
[illegible]wen's Odontography ............ 289
[illegible]wen on British fossils............ 539
P.
Paget on obstruction of the pulmonary arteries ................ 437
Palate, cleft, Fergusson on ......... 431
Paralysis, Copland on Pathology of.. 143
Paralysis, crossed ................ 143
Paralysis, relaxation of sphincters in, 146
Paralysis, treatment of ........... 188
Paralysis, intermission of...... 204, 238
Paralysis, temperature in .......... 420
Paralysis in animals ............. 475
Parasites, vegetable, of diseased structures ................... 247, 261
Parker on syphilis................ 454
Patholology, Heusinger's comparative 459
Peacock on obliteration of vena cava 191
Pelvis, the female ............... 517
Pelvis, deviations from the standard.. 519
Pericarditis, causes of............. 443
Pericarditis from rheumatism ...... 445
Pericarditis from Bright's disease.... 448
Peritonitis, treatment of ......... 50
Peritonitis, diagnosis of........... 52
Peritonitis after rheumatism........ 174
Peritonitis, temperature in......... 416
Pertussis, temperature in .......... 420
Pestilence, choleric.............. 147
Pharynx, abscess of ............. 225
Phlebitis, uterine ............... 177
Phlebitis, Lebert on ............. 240
Phthisis, rarity of, in Algeria ....67, 139
Phthisis in various climates ........ 138
Phthisis, change of climate in ...... 139
Phthisis, frequency in prisons ...... 214
Phthisis, temperature in ......... 419
Physiology, Lebert's pathological.... 235
Placenta, expulsion of ........... 521
Pleurisy, temperature in ......... 418
Pneumonia, temperature in ........ 418
Poisons, charcoal as an antidote for.. 332
Polypus-uteri ................ 355
Porrigo, vegetable parasites in ...... 247
Pregnancy, signs of ............. 562
Presentations, head, mechanism of .. 521
Prickly heat.................. 11
Prisons, Baly on the mortality of.... 208
Prisons, diet in ................ 212
Prisons, phthisis in.............. 214
Prisons, American .............. 215
Proteine, Prout on.............. 536
Prout's Bridgewater Treatise........ 535
Provincial Association, Transactions of 533
Puerperal fever, treatment of.... 175, 555
Puerperal ophthalmia.............. 437
Purulent infection ............ 176, 240
Pus, elimination of................ 176
Pus, characters of................ 237
Pustule, malignant................ 468
R.
Rabies, Heusinger on.............. 475
Raciborski on galvanism ..........5 53
Rainey on the structure of the lungs 451
Ratier on syphilis ............... 456
Rectum, fissure of the ........... 228
Red Sea, colour of the ........... 218
Rees on the blood and urine........ 150
Reichenbach on magnetism ........ 536
Reptiles, reno-portal vein of........ 403
Reptiles, pulsating lymphatic hearts of 403
Rheumatism, treatment of acute .... 60
Rheumatism distinguished from gout 90
Rheumatism, Schoenlein on .... 172, 189
Rheumatism, suppuration in........ 173
Rheumatism, aconite in... ....... 257
Rheumatism in India.............. 377
Rheumatism as a cause of pericarditis 445
Rheumatism, disease of the heart, in chronic ..................... 446
Rheumatism in animals............ 473
Rickets, temperature in............ 420
Robarts on laryngotomy .......... 324
Robertson on gout................ 78
Roger on temperature of children.... 408
Rot in sheep .................. 470
Roux on excision of joints ........ 545
S.
Sampson on homœopathy.......... 538
Scalp, contusion of ............... 223
Scalp, erysipelas of............... 224
Scarlatina, state of urinary organs in 184
Scarlatina, temperature in ........ 414
Scarlatina, Brown on.............. 540
Schirrhus, Walshe on .... ....... 479
Schoenlein's clinical lectures ...... 171
Sciatica, aconite in................ 256
Scleremis of infants .............. 422
Scorbutus in India............... 375
Scrofula in animals .............. 472
Scrofula in prisons............... 214
Skin, sympathy of with the liver.... 24
Skin, Wilson on the healthy........ 274
Skin, Trousseau on diseases in children 549
Skin, alkalis and caustics in diseases of 541
Skin-bound disease, the............ 422
Small-pox in the fœtus........ ....... 359
Smith on fruits and farinacea........ 378
South on hernia.................. 508
Southam's case of Ovariotomy...... 531
Speculum, uses of the ............ 339
Spinal cord, functions and lesions of.. 145
Spinal irritation, Griffin on ........ 53
Spinal sinuses, congestion of........ 145
Spine, curvature of, Coles on ...... 71
Spine, curvature of, prone position in 74
Spine, curvature of, Tamplin on .... 399
Spleen, hydatids of............... 267
Spleen disease in animals .......... 464

Sprains, galvanism in ............ 553
Stammering, Bishop on............ 322
Stanley on pulsating tumour of bones 433
Staphyloraphy, Fergusson on ...... 431
Stomach, diseases of, in animals .... 462
Stomach, perforation of............ 331
Strangulation, internal case of...... 330
Stricture, Levy and Lallemand on.... 97
Stricture, nature of .............. 101
Stricture, spasmodic .............. 102
Stricture, causes of .............. 104
Stricture, seat and number of ...... 105
Stricture, effects of ............. 106
Stricture, exploration of .......... 108
Stricture, treatment of ............ 109
Stricture, complications of ........ 117
Stricture in females .............. 342
Swan on the nerves of the uterus .. 536
Syphilitic eruptions .............. 232
Syphilis, Colles on the treatment of.. 231
Syphilis, combined with scrofula.... 233
Syphilis, secondary, Colles on ...... 234
Syphilis, Parker and Ratier on...... 454
Syphilis in animals................ 474

T.

Talipes equinus ....... .......... 390
Talipes varus ........ .......... 391
Talipes valgus.................... 393
Tamplin on deformities............ 388
Taylor on pericarditis............. 443
Teale on hernia .................. 508
Teeth, Owen on intimate structure of 291
Teeth, Owen on the formation of .. 296
Teeth, Hunter & Blake on formation of 300
Teeth, Owen on the variations of the 304
Temperatures of children .......... 408
Temperature in disease ........408, 424
Temperature, high post-mortem .... 427
Thompson on Hydatids............. 327
Tinea, vegetable parasites in........ 246
Todd on anatomy of the brain ...... 285
Trousseau on eruptions of children.. 549
Trusses, observations on .......... 513
Tubercle, characters of ............ 242
Tubercle, distinguished from cancer.. 499
Tubercle, temperature in ..... .... 419
Tulk's translation of Wagner ...... 401
Tumours of eyelids ............... 224
Tumours, classification of.... ..... 247
Tumours, distinguished from cancer 496
Tumour of the abdomen, case of.... 325

U.

Ulcers, old, treatment of ...... 358, 35[illegible]
Urea, detection and properties of.... 15[illegible]
Urethra, anatomy of .............. 9[illegible]
Urethra, muscularity of............ 10[illegible]
Urethra, calculi in the .......... 117
Urethra, dilatability of prostatic portion of....................... 363
Urethro-plastie, case of............ 440
Urinary abscess .................. 119
Urinary diseases, treatment of...... 160
Urinary fistulæ .................. 119
Urine, infiltration of . ............ 118
Urine, analysis of the.............. 154
Urine, solid contents of............ 159
Urine, examination of, in disease ... 184
Urine, retention of, Colles on ...... 230
Uteri, os, rigidity of .............. 180
Uteri, os, dilatation of, in labour .... 520
Uteri, cervix, ulceration of ........ 348
Uteri, cervix, chancre of .......... 351
Uteri, cervix, treatment of inflamed.. 351
Uteri, prolapsus.................. 343
Uteri, polypus .................. 348
Uterus, rheumatism of ............ 345
Uterus, disease in animals.......... 470
Uterus, nerves of ................ 527

V.

Vapour-bath, a cheap one.......... 563
Variola, temperature in............. 414
Variola in animals................ 467
Variola in the fœtus .... .. ...... 359
Variola, cauterizing .............. 542
Vegetables, nutritious powers of.... 380
Velpeau on Iodine Injections ...... 554
Vena-cava, obliteration of ......... 191
Vena-porta, inflammation of ....... 178
Venesection, aneurism after........ 358
Wagner's Comparative Anatomy.... 401
Waller on obstruction of power ..... 329
Walshe on cancer ................ 475
Webster on pathology of mental diseases ......................... 441
West on Cephalhæmatoma ........ 438
White-swelling, syphilitic.......... 229
White-swelling, Tamplin on........ 396
Williams on narcotics in insanity.... 287
Wilson on Echinococcous hominis .. 194
Wilson on healthy skin............ 274
Worms, intestinal, Klencke on...... 270
Worms, mode of introduction ...... 271
Wry-neck, Tamplin on ............ 400

### BIBLIOGRAPHICAL RECORD.

1. Elements of Materia Medica and Therapeutics. By Edward Ballard, M.D., Lond., & Alfred Baring Garrod, M.D., Lond. Octavo., pp. 487. London, 1845.

2. A Practical Treatise on Healthy Skin, with Rules for the Medical and Domestic Treatment of Cutaneous Diseases. By Erasmus Wilson, F.R.S. Small 8vo, pp. 380. London, 1845.

3. The Modern Treatment of Syphilitic Dieases, both primary and secondary, including numerous Formulæ for the Preparation and Mode of Administration of the New Remedies. By Langston Parker. Second Edition. Small 8vo, pp. 240. London, 1845.

*In our next.*

4. First Steps to Anatomy. By James Drummond, M.D., Professor of Anatomy and Physiology in the Belfast Institution. 12mo, plates, pp. 201. London, 1845.

5. On the Analysis of the Blood and Urine in Health and Disease, and on the Treatment of Urinary Diseases. By G. Owen Rees, M.D., F.R.S., &c. Second Edition. 8vo. pp. 228. London, 1845.

6. A System of Surgery. By J. M. Chelius. Translated by J. F. South. Parts 6, 7, and 8.

7. Medico-Chirurgical Transactions, published by the Royal Medical and Chirurgical Society of London. Second Series. Vol. X. Pp. 675. London, 1845.

8. Illustrations of Modern Mesmerism from Personal Investigation. By John Forbes, M.D., F.R.S. 18mo. pp. 113. London, 1845.

9. The Potatoe Disease, its Origin, Nature, and Prevention; with a Chemical, and Microscopical Analysis of the sound and diseased Tubers. By G. Phillips, of the Excise, and Member of the London Chemical Society. 12mo, plates, pp. 58. London, 1845.

*Contains a good deal of very useful and curious information on a subject which attracts much notice at present.*

10. Recherches de Pathologie Comparée Par Ch. F. Heusinger. Three Parts. 4to. Cassel, 1844.

11. Guy's Hospital Reports. Part III. October, 1845. S. Highley, Fleet Street.

12. The American Journal and Library of Dental Science. June, 1845. Edited by C. A. Harris, M.D., E. Maynard, M.D., and A. Westcott, M.D. Baltimore.

13. Report of the Medical Officers of the Lunatic Asylum for the County of Lancaster. Lancaster, 1841, 1845.

14. The Descriptive and Physiological Anatomy of the Brain, Spinal Cord, and Ganglions, and of their Coverings; adapted for the Use of Students. By Robert Bentley Todd, M.D. Post 8vo. pp. 284. London, 1845.

15. An Essay on the Use of Narcotics and other Remedial Agents, calculated to produce Sleep, in the Treatment of Insanity; for which the Author obtained the Lord Chancellor's Prize in Ireland. By Joseph Williams, M.D. Post 8vo, pp. 120. London, 1845.

16. A Clinical Introduction to the Practice of Auscultation and other Modes of Physical Diagnosis, intented to simplify the Study of the Diseases of the Lungs and Heart. By H. M. Hughes, M.D. 12mo, pp. 246. London, 1845.

17. An Inaugural Address, delivered at the Opening of the Norfolk and Norwich Hospital Museum, Sept. 10, 1845.

18. De Lithiasi Vesicæ Urinariæ in Genere, et in Specie, de extractione Calculi per Sectionem Perinæi, Dissertatio Medico-Chirurgica quam annuente Amplissimo Medicorum ordine in Universitate Caesarea Mosquensi pro gradu Doctoris Medicinæ et Chirurgiæ, Basilius Bassard. 4to, nine plates. Mosquæ, 1841.

19. Lectures on Natural and Difficult Parturition. By Edward Wm. Murphy, A.M., M.D., Professor of Midwifery, University College, London. 8vo, pp. 275.

*In our next.*

20. An Address by the Society of Apothecaries to the General Practitioners of England and Wales, on the Second Report of the Joint Deputation of the Society of Apothecaries, and the National Association of General Practitioners. 8vo, pp. 28. S. Highley, London.

21. The Buffalo Medical Journal. Edited by Austin Flint, M.D. Nos. 1 to 6, June to November. Buffalo.

22. The American Journal of the Medical Sciences. Edited by Isaac Hayes, M.D. No. 22, Oct. 1845. Philadelphia.

23. *Explanations*, a Sequel to Vestiges of the Natural History of the Creation. By the Author of that Work. Small 8vo, pp. 198. London, 1845.

24. A Supplement to the History of British Birds. By William Yarrell, F.L.S., &c. 8vo, pp. 53. London, 1845.

25. Observations on the Nosological Arrangement of the Bengal Medical Returns, with Cursory Remarks on Medical Topography and Military Hygiene. By Fred. J. Monat, M.D., &c. Calcutta, 1845. 8vo, pp. 64.

26. A Memoir on Amputation of the Thigh at the Hip-Joint, with a successful Case. By William Sands Cox, F.R.S. &c.

27. On Scarlatina, and its successful Treatment, by the Acidum Aceticum Dilutum of the Pharmacopœia. By J. B. Brown. Small 8vo, pp. 66. London, 1845.

28. An Act (8 & 9 Vict., c. 100) for the Regulation of the Care and Treatment of Lunatics. With Explanatory Notes and Comments, &c. By Forbes Winslow, M.D. Small 8vo, pp. 173. London, 1845.

*Most useful alike to the legal and medical profession.*

29. The Veterinarian. Nos. for October, November, and December.

30. An Address at the Opening of the Classes of the Medical School attached to the Middlesex Hospital, Oct. 1845. By Alexander Shaw, Esq.

*A remarkably well-written Address; the illustrations are good, and the language terse and most expressive.*

